# Alcohol consumption in tertiary education students

**DOI:** 10.1186/1471-2458-11-545

**Published:** 2011-07-09

**Authors:** Nicola J Reavley, Anthony F Jorm, Terence V McCann, Dan I Lubman

**Affiliations:** 1 Orygen Youth Health Research Centre, Centre for Youth Mental Health, University of Melbourne, Locked Bag 10, Parkville, Victoria 3052, Australia; 2 School of Nursing and Midwifery, Victoria University, PO Box 14428, Melbourne, Victoria 8001, Australia; 3 Turning Point Alcohol and Drug Centre, Eastern Health and Monash University, 54-62 Gertrude Street, Fitzroy, Victoria 3065, Australia

## Abstract

**Background:**

Heavy alcohol consumption among adolescents and young adults is an issue of significant public concern. With approximately 50% of young people aged 18-24 attending tertiary education, there is an opportunity within these settings to implement programs that target risky drinking. The aim of the current study was to survey students and staff within a tertiary education institution to investigate patterns of alcohol use, alcohol-related problems, knowledge of current National Health and Medical Research Council (NHMRC) guidelines for alcohol consumption and intentions to seek help for alcohol problems.

**Methods:**

Students of an Australian metropolitan university (with staff as a comparison group) participated in a telephone interview. Questions related to knowledge of NHMRC guidelines, drinking behaviour, alcohol-related problems and help-seeking intentions for alcohol problems. Level of psychological distress was also assessed.

**Results:**

Of the completed interviews, 774 (65%) were students and 422 (35%) were staff. While staff were more likely to drink regularly, students were more likely to drink heavily. Alcohol consumption was significantly higher in students, in males and in those with a history of earlier onset drinking. In most cases, alcohol-related problems were more likely to occur in students. The majority of students and staff had accurate knowledge of the current NHMRC guidelines, but this was not associated with lower levels of risky drinking. Psychological distress was associated with patterns of risky drinking in students.

**Conclusions:**

Our findings are consistent with previous studies of tertiary student populations, and highlight the disconnect between knowledge of relevant guidelines and actual behaviour. There is a clear need for interventions within tertiary education institutions that promote more effective means of coping with psychological distress and improve help-seeking for alcohol problems, particularly among young men.

## Background

Heavy alcohol consumption among adolescents and young adults is an issue of significant political and public health concern. Indeed, analysis of data from the 2007 *Australian National Survey of Mental Health and Wellbeing *(NSMHWB) demonstrates that substance use disorders (but particularly harmful use of alcohol) are the most common mental disorders among young men (aged between 16 and 24), with a higher prevalence than both anxiety and affective disorders combined [1]. While young people are less likely than older people to seek help for mental disorders, this gap is particularly notable for young men with substance use disorders [1].

Australian National Health Surveys have shown that levels of risky drinking in those aged 18-24 years have increased since 1995 [2]. Although the picture is complex, there is some evidence that rates of alcohol-related harm among young people have also increased significantly over recent years [3,4]. However, despite higher rates of harmful use, many young people minimise the risks associated with risky drinking and often view excessive alcohol consumption as a culturally normal in their age range [4,5].

The results of these surveys suggest the need for population health approaches that tackle young people's erroneous beliefs about alcohol consumption and related harms. With approximately 50% of young Australians aged between 18 and 20 years in tertiary education [6], such educational institutions provide a unique environment for interventions as they encompass several aspects of students' lives, including educational activities, health services, residences, social networks and extracurricular activities.

Alcohol is the greatest single contributor to college student illness and death in the US [7]. In a study of over 2500 New Zealand university students, 81% of students drank in the previous four weeks, 37% reported one or more binge episodes in the last week, and 68% drank to hazardous levels [8]. While students tend not to drink every day, they are more likely to drink at risky levels when they do drink [9]. They typically drink more at weekends, early in a semester and during university breaks, but less during exams [10]. However, much of the research in this area has been carried out in the US and, while the available evidence suggests that the pattern is similar, relatively limited comparable data is available for Australian tertiary students [11-13]. It is likely that cultural differences, as well as differences in the legal drinking age may affect levels of alcohol consumption among tertiary students.

In order to design appropriate interventions for use in tertiary education settings, there is a need to further investigate Australian students' drinking patterns as well as knowledge of and attitudes towards drinking, and help-seeking. The aim of this paper is to investigate alcohol consumption, alcohol-related problems, intentions to seek help for alcohol problems and knowledge of National Health and Medical Research Council (NHMRC) guidelines for alcohol consumption in a group of students at a tertiary education institution. The current NHMRC guidelines, which were published in 2009, recommend drinking no more than two standard drinks a day to reduce the lifetime risk of harm from alcohol-related disease or injury. They also recommend drinking no more than four standard drinks on a single occasion to reduce the risk of alcohol-related injury arising from that occasion. Data was also collected from staff using the same methods to enable direct comparison, enabling the identification of drinking patterns, knowledge and attitudes that are particular to students.

## Methods

### Participants

Participants consisted of staff and students from Victoria University in metropolitan Melbourne, Australia. Victoria University has several campuses and offers a broad mix of courses, including a large number of vocational education (VE) courses (including technical and trades), as well as higher education (HE) courses. Staff and students were invited to participate through emails and through postcards handed out by researchers. Participants were able to indicate agreement to participate via a website or by returning the postcards to the researchers. Those that did so were contacted for participation in a computer-assisted telephone interview between March and May 2010. The overall response rate was 95%, defined as completed interviews (1197) out of sample members who returned contact details and were confirmed as in scope (1265).

### Interview content

Interviews were based around a vignette of a 21 year-old person with depression (30-year old in staff vignette). Respondents were asked questions about their sociodemographic characteristics, recognition of the disorder in the vignette, what they would do to seek help if they had the problem, beliefs and intentions about first aid, beliefs about interventions, stigmatising attitudes, exposure to mental disorders and their level of psychological distress using the Kessler 6 (K6). The K6 is a six-item screening scale with strong psychometric properties that is able to discriminate DSM-IV cases from non-cases and has been widely used in general-purpose health surveys [14]. Reliability as assessed by Cronbach's alpha for the K6 in this study was 0.81. Participants were also asked about their knowledge of current NHMRC guidelines for alcohol consumption (defined as no more than two drinks per day to reduce lifetime risk of harm, and no more than four drinks on any one occasion to reduce risk of injury arising from that occasion) [15].

Those who had drunk alcohol at some point in their lives were administered the Alcohol Use Disorders Identification Test (AUDIT) [16]. The AUDIT is a 10-item questionnaire designed to screen for early-stage problem drinking. It is widely used and has been validated in tertiary student populations [17]. Reliability as assessed by Cronbach's alpha for the AUDIT in this study was 0.65. Participants who regularly drank alcohol were asked about past 6-month problems arising as a result of alcohol consumption (questionnaire adapted from McGee and Kypri [18]) and help-seeking intentions for alcohol problems.

### Statistical analysis

Descriptive data on respondents was analysed using percent frequencies and 95% confidence intervals, with the sample divided into staff and students. Multiple logistic regressions were used to examine the association between respondent characteristics and AUDIT scores and between drinking patterns, consequences of drinking, knowledge and K6 scores. For the first regression, AUDIT scores were dichotomised into low risk (7 or lower) or medium/high risk categories (8 or above), and variables representing respondent characteristics were entered simultaneously as predictors. These included age (in years), gender, level of education (bachelor degree or above vs. other), country of birth (Australia vs. other), employment status (not working vs. working), K6 scores, age of first alcohol consumption, awareness of NHMRC guidelines and accuracy of knowledge of these (nomination of number of drinks within guidelines vs. above guidelines). The latter three covariates were coded into three categories: awareness/correct knowledge of guidelines, lack of awareness/incorrect and don't know/missing. For the second regression, K6 scores were dichotomised into low risk (14 or lower) and moderate/high risk (15 or higher). All analyses were performed using PASW Version 18.

### Ethics

This study was approved by Victoria University Human Research Ethics Committee.

## Results

Of the completed interviews 774 (65%) were students and 422 (35%) were staff. This represents 2% and 17% of the total students and staff at targeted campuses respectively. As shown in Table 1, mean (SD) ages of students and staff were 24.5 (8.4) and 44.4 (11.2) years respectively. Age ranges of student survey participants were compared with those of all Victoria University students and were found to be significantly different (χ^2 ^(6) = 33.35, p < 0.001). However, modal age range was age 20-29 in both samples. Relative to the whole university population, the survey sample was under-represented in the under 18 and over-represented in the over 40 age groups. Over 60% of respondents in each category were female (compared to 50% in the whole university population) and over 85% were Australian citizens. Among students, the majority (87%) were studying full time, with over half studying for a bachelor degree (compared to 42% of the total student population). Over 60% were working in some capacity. The majority of staff (62%) were full time employees.

**Table 1:** Sociodemographic characteristics of participants

	Students (n = 774)	Staff (n = 422)
Age (mean (SD))	24.5 (8.4)	44.4 (11.2)
*Gender*		
Male	38%	34%
Female	62%	66%
*Citizenship*		
Australian citizens	86%	93%
Country of birth Australia	71%	74%
*Education*		
Studying full time	87%	-
Bachelor degree	55%	-
Diploma	18%	-
Other	27%	-
*Employment*		
Full time	10%	62%
Part time	20%	14%
Casual	31%	9%
Not working	38%	-
Looking for work	24%	-
Contract	0	15%

### Alcohol consumption and knowledge of NHMRC guidelines

AUDIT scores, alcohol use and knowledge of NHMRC guidelines are given in Table 2. Staff were more likely to drink regularly, with 70% drinking twice per month or more compared to 48% of students, but students were more likely to drink heavily, with 33% drinking 6 or more drinks in one session at least monthly, compared to 21% of staff. Male students and staff were significantly more likely than females to drink 6 drinks per session monthly or more. However, staff or students in the moderate/high risk K6 group were not more likely to drink 6 drinks per session monthly or more. Mean AUDIT scores were significantly higher for students than staff (t(898) = 3.05, p < 0.01), for male students compared to female students (t(443) = 3.31, p = 0.001) and male staff compared to female staff (t(392) = 2.35, p = 0.019). Students were more likely than staff to be in the medium risk category and less likely to be in the low risk category. However, a greater number of students than staff were non-drinkers. Age of first alcohol consumption (other than a few sips) was significantly lower in students than staff (t(619) = -4.49, p < 0.001). Beer, wine and spirits were the most commonly consumed types of alcohol, with students most likely to drink spirits and staff most likely to drink wine.

**Table 2:** Alcohol consumption and knowledge of NHMRC guidelines

	Students (n = 774)	Staff (n = 422)
Mean (SD) AUDIT score (females)	5.4 (5.2)	4.5 (4.3)
Mean (SD) AUDIT score (males)	7.0 (6.4)	5.6 (4.5)
Mean (SD) AUDIT score (all)	6.0 (5.7)	5.0 (4.5)
Drink alcohol ≥ twice per month	48% (95% CI 45-51)	70% (95% CI 66-74)
Drink ≥ 6 drinks per session monthly or more (females)	29% (95% CI 26-32)	16% (95% CI 13-20)
Drink ≥ 6 drinks per session monthly or more (males)	39% (95% CI 36-42)	30% (95% CI 26-34)
Drink ≥ 6 drinks per session monthly or more (all)	33% (95% CI 30-36)	21% (95% CI 18-24)
*Risk of alcohol related harm*		
Low risk (≤ 7)	58% (95% CI 55-61)	75% (95% CI 71-79)
Medium risk (8-15)	20% (95% CI 17-23)	14% (95% CI 11-17)
High risk (≥ 16)	6% (95% CI 4-8)	4% (95% CI 2-6)
Never drank alcohol	15% (95% CI 12-18)	7% (95% CI 5-9)
Age of first alcohol - mean (SD)	16.1 (2.8)	17.2 (3.5)
*Most commonly drunk types of alcohol*		
Beer (full strength)	24% (95% CI 21-27)	12% (95% CI 9-15)
Wine	20% (95% CI 17-23)	59% (95% CI 54-64)
Spirits	46% (95% CI 42-50)	10% (95% CI 7-13)
*NHMRC guidelines*		
Aware of guidelines	65% (95% CI 62-68)	79% (95% CI 75-83)
Nomination of number of drinks a day to reduce the risk to long term health		
Within guidelines (≤ 2)	77% (95% CI 74-80)	82% (95% CI 78-86)
Greater than guidelines (≥ 3)	12% (95% CI 10-14)	10% (95% CI 7-13)
Don't know	11% (95% CI 9-13)	8% (95% CI 6-11)
Nomination of number of drinks on one occasion to reduce the risk of injury		
Within guidelines (≤ 4)	58% (95% CI 54-61)	63% (95% CI 58-68)
Greater than guidelines (≥ 5)	29% (95% CI 26-32)	22% (95% CI 18-26)
Don't know	13% (95% CI 11-15)	14% (95% CI 11-17)

The majority of both students and staff were aware of the NHMRC guidelines relating to alcohol consumption, although staff were more likely to be aware than students (79% vs 65% respectively). Awareness of the recommended maximum number of drinks a day required to avoid long term health risks was more common than that of the maximum number of drinks on any occasion required to reduce the risk of injury. Those in the moderate/high K6 group were significantly less likely to have heard of the guidelines.

### Alcohol-related problems and help seeking

Table 3 outlines the alcohol-related problems experienced in the previous six months by those who drank alcohol regularly. Almost all problems were more likely to occur in students than staff. Hangovers were the most common problems and were experienced by 62% of students and 36% of staff. Twenty eight percent of students reported being sick or having passed out, and over 20% had either an emotional outburst or an argument when drinking. Twelve percent of students said they did less well in their studies as a result of their alcohol consumption. When male and female students were compared, male students were more likely to have sex which they were unhappy about at the time (χ^2 ^(1) = 5.24, p = 0.022), steal property (χ^2 ^(1) = 4.44, p = 0.035), commit acts of vandalism (χ^2 ^(1) = 24, p < 0.001) or be asked to leave a party, pub or club (χ^2 ^(1) = 8.39, p = 0.004). Among staff, males were more likely to have an accident (χ^2 ^(1) = 4.01, p = 0.045) or be asked to leave a party, pub or club (χ^2 ^(1) = 8.06, p = 0.005) and have sex which they were unhappy about at the time (χ^2 ^(1) = 4.01, p = 0.045). No other gender differences were observed.

**Table 3:** Alcohol-related problems experienced in previous 6 months

	Students % (95% CI)			Staff % (95% CI)		
	**Male (n = 212)**	**Female (n = 354)**	**Total (n = 566)**	**Male (n = 120)**	**Female (n = 239)**	**Total (n = 359)**

Hangover	65 (60-70)	61 (57-65)	62 (58-66)	38 (31-45)	33 (28-38)	35 (31-39)
Sick/passed out	28 (23-33)	28 (24-32)	28 (24-32)	3 (1-6)	5 (3-7)	5 (3-7)
Emotional outburst	18 (14-22)	25 (21-29)	22 (19-25)	8 (4-12)	12 (9-15)	11 (8-14)
Argument	21 (16-26)	21 (17-25)	21 (18-24)	7 (3-11)	6 (3-9)	6 (4-8)
Do less well in studies	12 (8-16)	11 (8-14)	12 (9-15)	-	-	-
Sex later regretted	10 (7-13)	7 (5-9)	8 (6-10)	3 (0-6)	0	1 (0-2)
Asked to leave^a, b^	11 (7-15)	5 (3-7)	7 (5-9)	3 (0-6)	0	1 (0-2)
Trouble at home or work	6 (3-9)	4 (2-6)	5 (3-7)	4 (1-7)	2 (1-3)	3 (2-4)
Accident^b^	7 (4-10)	6 (4-8)	6 (4-8)	2 (0-4)	0	1 (0-2)
Violent	9 (6-12)	5 (3-7)	6 (4-8)	0	0	0
Sex unhappy about at time^a, b^	9 (6-12)	4 (2-6)	6 (4-8)	2 (0-4)	0	1 (0-2)
Steal property^a^	5 (3-7)	2 (1-3)	3 (2-4)	0	0	0
Vandalism^a^	8 (5-11)	0	3 (2-4)	0	0	0
Depressed if unavailable	5 (3-7)	2 (1-3)	3 (2-4)	1 (0-3)	5 (3-7)	3 (2-4)

^a ^significantly higher in male students; ^b ^significantly higher in male staff.

Several alcohol-related problems were more likely to occur in students with moderate or high K6 scores compared to those with low K6 scores. These included emotional outbursts, doing less well in their studies, having trouble at home, having arguments, having sex about which they were unhappy at the time, having sex which they later regretted and becoming depressed if alcohol was unavailable. For staff, there were no differences in alcohol-related problems according to whether a person was in the low or moderate/high risk group.

The great majority of students (88%) and staff (91%) said they would seek help for alcohol problems (see Table 4). GPs were the most commonly mentioned sources of help. However, only 25% of students said they would go to a GP while over 50% of staff reported they would.

**Table 4:** Help seeking for alcohol misuse

	Students (n = 566) % (95% CI)	Staff (n = 330) % (95% CI)
Yes - would seek help for alcohol problem	88 (85-91)	91 (88-94)
*Source of help*		
GP	25 (21-29)	53 (48-58)
Drug and alcohol service	20 (17-23)	22 (18-26)
Counsellor	13 (10-16)	8 (5-11)
Friend	12 (9-15))	4 (2-6)
Parents	11 (8-16)	2 (0-4)
Family member	7 (5-9)	5 (3-7)
Helpline	5 (3-7)	3 (1-5)
VU counsellor	4 (2-6)	-
Employee Assistance Program	-	4 (2-6)
Psychologist	3 (2-4)	3 (1-5)
Psychiatrist	1 (0-2)	1 (1-2)
Lecturer	0	-
Supervisor	-	0
Co-worker	-	0

### Factors associated with risky drinking

As shown in Table 5, binary logistic regression analyses revealed that factors associated with medium/high risk AUDIT scores in students were male gender, being born in Australia, higher K6 score and a younger age of first alcohol consumption. Among staff, the only factor associated with higher AUDIT scores was a younger age of first alcohol consumption. There was no association with AUDIT scores in either staff of students for age, education level, employment status, awareness of NHMRC guidelines or the nomination of the number of drinks a day or on any one occasion within the guidelines.

**Table 5:** Factors associated with risky drinking

	Students Medium or high risk AUDIT scores OR 95% CI	Staff Medium or high risk AUDIT scores OR 95% CI
Age	0.98 (0.95-1.00)	0.98 (0.95-1.01)
Female gender	0.49 (0.32-0.75)*	0.75 (0.42-1.36)
Country of birth other than Australia	0.35 (0.20-0.64)*	0.52 (0.25-1.09)
Education level (bachelor degree/postgraduate vs diploma/certificate)	0.77 (0.51-1.15)	-
Employment status: not working vs working	1.09 (0.72-1.64)	-
K6 total score	1.06 (1.01-1.11)*	1.05 (0.98-1.14)
Age of first alcohol consumption	0.73 (0.65-0.80)***	0.84 (0.76-0.93)**
Awareness of NHMRC guidelines		
Aware vs not aware	1.18 (0.78-1.79)	0.77 (0.37-1.59)
Aware vs don't know	0	0
Nomination of number of drinks per day relative to guidelines		
Within vs above	1.50 (0.85-2.66)	1.94 (0.84-4.49)
Within vs don't know	0.52 (0.23-1.20)	0.90 (0.23-3.49)
Nomination of number of drinks on one occasion relative to NHMRC guidelines		
Within vs above	1.53 (0.99-2.33)	1.29 (0.69-2.41)
Within vs don't know	1.69 (0.89-3.19)	1.02 (0.38-2.78)

Legend: *p < 0.05; **p < 0.01; ***p < 0.001.

## Discussion

The aim of the study was to investigate alcohol-related knowledge and attitudes and risky behaviours in students and staff of an Australian university. When compared to staff, students were more likely to drink alcohol at risky levels, with 26% of students in the medium or high risk categories compared to 18% of staff. Mean AUDIT scores were significantly higher and mean age of first alcohol consumption was lower in students. In almost all cases, alcohol-related problems were more likely to occur in students than staff. The majority of students and staff were aware of the NHMRC guidelines for alcohol consumption and had accurate knowledge of these. However, this knowledge was not associated with a lower likelihood of risky alcohol consumption in either staff or students.

The results of this study are broadly in keeping with both Australian and international studies suggesting that tertiary education students, particularly males, have relatively high levels of risky alcohol consumption [19,20]. In the current study, while students were less likely to drink regularly, they were more likely to drink heavily when they did consume alcohol. In a study of 400 Queensland students, Roche and Watt [13] found that 94% drank alcohol and 54% drank five or more drinks on a typical drinking occasion. Another study of 275 Australian students, revealed that 88% of students drank alcohol, with 45% drinking weekly and over 40% drinking five or more drinks in a single session [11].

Age of first alcohol consumption was significantly lower in students than staff and may be seen in the context of a generational shift towards earlier alcohol consumption [4]. As noted in Table 5, age of first alcohol consumption was associated with risky drinking. This is consistent with other evidence linking early onset drinking with increased risk of developing later alcohol use disorders [21,22]. In the current study, the mean age of first alcohol consumption was 16.1 years, which is reasonably consistent with other Australian data [23]. The most popular type of alcohol consumed by students in the current study was spirits, whereas staff were most likely to drink wine. This supports previous research that has shown that young people are most likely to drink bottled spirits, liqueurs and pre-mixes in cans and bottles and that consumption of these drinks has increased in recent years, particularly among 15-17 year olds [24]. There is some evidence that alcohol consumption is higher in Australian adolescents and young adults than in other countries, notably the US, pointing to the importance of gathering Australian data [25,26].

Knowledge of NHMRC guidelines for daily alcohol consumption was reasonably accurate, with over 75% of both students and staff able to nominate a number within the guidelines. These rates are higher than those reported in other studies. The 2004 National Drug Strategy Household Survey found that approximately 40% of 18-24 year-olds had heard of the guidelines [4]. Hasking et al. [27] found that over 50% of students overestimated the number of drinks recommended in the guidelines. Knowledge of the number of drinks on one occasion required to reduce the risk of injury was less accurate, with 58% of students and 63% of staff getting this correct. This may be partly explained by the relatively recent changes in these guidelines, which, prior to 2009, advised men to drink no more than six standard drinks in one day and women four. Analysis of the factors associated with risky alcohol consumption revealed that accurate knowledge of guidelines was not associated with a lower likelihood of risky alcohol consumption in either staff or students. The 2009 NHMRC guidelines attracted criticism from some experts and members of the public, on the grounds that the new limits would be perceived as out of step with community standards and risked being ignored. The current data highlight the need for further research examining how to improve the effectiveness of the guidelines in changing drinking behaviour.

In those who did drink alcohol, the frequency of almost all alcohol-related problems was greater in students than staff and in males. In students and staff, hangovers were the most common problem and around 25% of students had been sick or had an emotional outburst in the previous six months. Male staff and students were more likely to have had sex which they were unhappy about at the time or had been asked to leave a party, pub or club. Male students were more likely to steal property or commit acts of vandalism. Other studies of adolescents and young adults have found similar rates of alcohol-related problems and some similar gender differences in the types of problems experienced [13,28,29]. In a study of New Zealand students, McGee and Kypri [18] also found that male students were more likely to steal, commit acts of vandalism or be asked to have a pub or club. For students but not staff, those with higher psychological distress appeared to suffer more negative consequences of alcohol consumption. These results suggest that interventions aimed at reducing the risk of harms associated with excessive drinking should address ways of coping with psychological distress and be tailored according to gender.

The great majority of respondents (88% of students and 91% of staff) reported that they would seek help if they had an alcohol problem, with preferred sources of help external to the institution. This contrasts with evidence documenting that help-seeking for alcohol problems is very low, particularly among young males [1]. This discrepancy is likely to be due to the low level of recognition of alcohol problems by those with the problems, as well as the stigma associated with having an alcohol problem, and points to the need for education campaigns to incorporate realistic and consistent messages about moderate alcohol intake and the professional help available [5]. While GPs were the most commonly cited sources of help by staff and students, only 25% of students and 53% of staff said they would go to a GP. Such results provide support for calls for policies and programs that improve help-seeking for alcohol problems, particularly among young men [30].

When factors associated with risky alcohol consumption were considered, those born in a country other than Australia were about half as likely to drink at risky levels. This may be explained by the relatively large number of such students from Asian cultures where young people are less likely to drink heavily [20,31].

Psychological distress was also associated with drinking at risky levels in students, although not in staff. It is well documented that depression, anxiety and alcohol misuse often occur together [32]. Alcohol may be used to help cope with depression and anxiety disorders and may worsen these disorders [33,34]. Brener *et al*. [35] found that when controlling for demographic characteristics, students who had considered suicide were at increased odds of using tobacco, alcohol, and illicit drugs.

Limitations of the study include the relatively mature age of students (mean age 24.5) and over-representation in the over 40 age group of students, a group who may have a greater interest in participating in the study. As such, this may limit the generalisability of the study findings to other tertiary institution populations, although many of the findings are consistent with other studies of tertiary students. Further limitations may arise from the self-report nature of the data, particularly concerning alcohol consumption. The AUDIT asks respondents to estimate the number of standard drinks they have consumed and there is evidence that many drinkers underestimate their alcohol consumption [27]. In addition, the cross-sectional nature of the data means that causal inferences about the direction of the associations between some of the factors listed in Table 5 and risky drinking cannot be drawn.

Results of the current study support the need for interventions that target alcohol misuse within tertiary education settings. Such interventions should focus on binge drinking and on the negative consequences of importance to students, such as the effect on grades. Poor knowledge of the NHMRC guidelines also points to the need to promote knowledge of the number of drinks on one occasion likely to reduce the risk of harm. The associations between psychological distress and risky drinking and negative consequences of alcohol consumption in students point to the need for interventions for alcohol misuse that aim to improve overall mental health literacy and promote more effective means of coping with psychological distress [36].

A recent US three-year multi-site study found that social norms marketing campaigns can be an effective component of campus efforts to reduce heavy drinking among first-year students, especially if implemented when students arrive on campus [37]. Some evidence suggests that focusing on events typically associated with high alcohol consumption, such as 21^st ^birthday parties and holidays, may also be beneficial [38-40]. Online interventions may have a role to play, with evidence supporting the effectiveness of personalised feedback interventions for alcohol misuse in tertiary education students [41-43].

## Conclusions

The results of this study suggest that tertiary education students, particularly males, have relatively high levels of risky alcohol consumption. In almost all cases, alcohol-related problems were more likely to occur in students than staff. The majority of students and staff in the study were aware of the NHMRC guidelines for alcohol consumption and had accurate knowledge of these. However, this knowledge was not associated with a lower likelihood of risky alcohol consumption in either staff or students and there is a need for further research on the links between knowledge of guidelines and drinking behaviour. Those who started drinking regularly at an early age were more likely to drink at risky levels, providing further support for approaches that delay the age of alcohol consumption among adolescents. With approximately 50% of young people aged between 18 and 24 in vocational or higher education, interventions in these institutions have the potential to play a substantial role in reducing risky drinking among this age group. Such interventions should aim to target the negative aspects of binge drinking, improve knowledge of NHMRC guidelines, promote more effective means of coping with psychological distress and improve help-seeking for alcohol problems, particularly among young men.

## Competing interests

The authors declare that they have no competing interests.

## Authors' contributions

NJR, AFJ and TVM participated in the design of the study and all authors participated in the questionnaire design. NJR carried out the data analysis and drafted the manuscript. AFJ supervised data analysis and provided comments on the manuscript. TVM and DIL provided comments on the manuscript. All authors read and approved the final manuscript.

## Pre-publication history

The pre-publication history for this paper can be accessed here:

http://www.biomedcentral.com/1471-2458/11/545/prepub
